# A multi-institution evaluation of deformable image registration algorithms for automatic organ delineation in adaptive head and neck radiotherapy

**DOI:** 10.1186/1748-717X-7-90

**Published:** 2012-06-15

**Authors:** Nicholas Hardcastle, Wolfgang A Tomé, Donald M Cannon, Charlotte L Brouwer, Paul WH Wittendorp, Nesrin Dogan, Matthias Guckenberger, Stéphane Allaire, Yogish Mallya, Prashant Kumar, Markus Oechsner, Anne Richter, Shiyu Song, Michael Myers, Bülent Polat, Karl Bzdusek

**Affiliations:** 1 Department of Human Oncology, University of Wisconsin-Madison, Madison, WI, USA; 2 Department of Physical Sciences, Peter MacCallum Cancer Centre, Locked Bag 1 A’Beckett St., Melbourne, VIC, 8006, Australia; 3 Centre for Medical Radiation Physics, University of Wollongong, Wollongong, NSW, Australia; 4 Departments of Medical Physics and Biomedical Engineering, University of Wisconsin-Madison, Madison, WI, USA; 5 Department of Radiation Oncology, University Medical Center Groningen, University of Groningen, Groningen, the Netherlands; 6 Department of Radiation Oncology, Virginia Commonwealth University Medical Center, Richmond, VA, USA; 7 Department of Radiation Oncology, University Hospital Würzburg, Würzburg, Germany; 8 Radiation Medicine Program, Princess Margaret Hospital, Toronto, ON, Canada; 9 Philips Electronics India Pvt. Ltd., Philips Innovation Campus, Bangalore, India; 10 Philips Radiation Oncology Systems, Madison, WI, USA

**Keywords:** Deformable image registration, Adaptive radiotherapy, Head and neck cancer

## Abstract

**Background:**

Adaptive Radiotherapy aims to identify anatomical deviations during a radiotherapy course and modify the treatment plan to maintain treatment objectives. This requires regions of interest (ROIs) to be defined using the most recent imaging data. This study investigates the clinical utility of using deformable image registration (DIR) to automatically propagate ROIs.

**Methods:**

Target (GTV) and organ-at-risk (OAR) ROIs were non-rigidly propagated from a planning CT scan to a per-treatment CT scan for 22 patients. Propagated ROIs were quantitatively compared with expert physician-drawn ROIs on the per-treatment scan using Dice scores and mean slicewise Hausdorff distances, and center of mass distances for GTVs. The propagated ROIs were qualitatively examined by experts and scored based on their clinical utility.

**Results:**

Good agreement between the DIR-propagated ROIs and expert-drawn ROIs was observed based on the metrics used. 94% of all ROIs generated using DIR were scored as being clinically useful, requiring minimal or no edits. However, 27% (12/44) of the GTVs required major edits.

**Conclusion:**

DIR was successfully used on 22 patients to propagate target and OAR structures for ART with good anatomical agreement for OARs. It is recommended that propagated target structures be thoroughly reviewed by the treating physician.

## Background

Modern radiation therapy has the ability to utilize multi-modality imaging technologies for disease definition, patient setup and treatment assessment. Daily image guidance using volumetric imaging has shown that anatomy revealed in the original planning CT scan often changes during treatment due to various causes including patient weight loss, tumor shrinkage, systematic motion (such as breathing) and random motion [1,2]. The effect of anatomical changes during the treatment course is that the original treatment plan may not provide necessary target coverage and organ at risk (OAR) sparing [1,3-5]. Adaptive radiotherapy (ART) aims to observe anatomical changes during the treatment course using volumetric imaging modalities and adjust the treatment plan when the plan quality degrades [6-11]. ART can be a time-consuming process, as target volumes and OARs must be delineated on the most recently acquired volumetric images to observe changes in doses [10].

Various methods to increase the speed of Region of Interest (ROI) delineation are used clinically, including atlas segmentation, ROI propagation (copying previous ROIs and editing manually) and Deformable Image Registration (DIR) [10,12-15]. DIR is the spatial mapping of corresponding locations (if they exist) between images and can be used for ROI delineation on a second image when there exists a set of ROIs on the first image. An advantage of DIR in ART is that the Deformation Vector Field (DVF) can then be used for non-rigid dose accumulation.

Safe and effective adaptive radiotherapy relies upon accurate, up-to-date ROIs. Brouwer et. al. [16] showed that computerized re-contouring of head and neck OARs is a useful alternative to physician re-contouring. However, Voet et. al. [15] showed that editing of Atlas-based auto-segmented ROIs is necessary to ensure sufficient target coverage in head and neck patients. Tsuji et. al. [17] showed that inaccurate automatic propagation of target structures lead to inferior dosimetric coverage in adaptive radiotherapy. It is beneficial that a DIR algorithm provides clinically acceptable propagated ROIs to reduce time and resources required for contour reviewing and correction in the ART process.

This study investigates the clinical acceptability of two mathematically different DIR algorithms for ROI propagation. Five institutions provided data to assess the agreement of DIR-propagated ROIs with expert physician drawn ROIs. ROIs were quantitatively compared using ROI comparison metrics. DIR-propagated ROIs were also reviewed and scored by expert physicians based on the level of correction required and the clinical utility of the propagated ROIs.

## Methods

Two DIR algorithms were evaluated for head and neck ART cases. The DIR algorithms and ROI propagation workflow were implemented in a research version of the Pinnacle^3^ Radiotherapy Planning System (v9.100, Philips Healthcare, Fitchburg, WI, USA). The algorithms used were Demons and Salient-Feature-Based Registration (SFBR). All deformations were performed on a clinical 16-core Sun Fire 4450 system. Both algorithms had numerous multi-threaded steps.

The Fast Symmetric Demons algorithm as implemented in the ITK toolkit was used [18]. Demons uses image intensity values and the assumption that pixels representing the same anatomical point on each image have the same image intensity values. Thus image intensity histogram matching is required prior to deformation. A regular grid of forces deforms the iso-intensity contours in the image using forces derived from the optical flow equation. A multi-resolution Demons technique was used in this study in which a maximum of 200, 100, 100 & 30 iterations were run at levels 4, 3, 2, & 1 respectively. Levels 4 through 1 are performed at 4X to 1X the CT grid resolution. A stopping criterion (set at 1.5%) is determined as the percentage change in mean square difference in intensities between the target image volume and deforming source volume for approximately 4 to 5 successive iterations. This allows enough iterations to be performed to reach convergence, yet terminates registration before the point beyond which computational effort is wasted. Therefore, the actual number of iterations was less than the maximum. The histograms for both images were matched prior to running DIR using 64 bins and 7 match points. The DVF was smoothed after each iteration using a Gaussian kernel with a standard deviation of 3, 3, 0.9 and 0.7 for levels 4 through 1. The parameters used for Demons DIR were initially varied and tested on a subset of the patients to determine the optimum values of each parameter. For this work, the Demons DVF is defined in the target image frame of reference. To obtain the non-rigidly propagated ROI on the target image, the algorithm cycles through each voxel in a sub-volume of the fixed image and obtains the ROI binary mask value in the corresponding voxel in the source image.

Salient-Feature-Based Registration (SFBR) uses 'salient features', that is, sharply prominent and distinctive features in the image [19]. It is a point-based registration approach using the automated equivalent of anatomical landmarks. The features are extracted in one image using an interest point detector and are assigned a center location as well as a scale. The feature locations are updated in the next image one-by-one independently by maximizing local intensity correlation given the feature scale. A feature is discarded as being unreliable if no correlation above 0.80 can be achieved within a search region. The salient feature locations in correspondence are then used as anchor points to interpolate a non-rigid transformation using the Thin Plate Splines (TPS) method. Typically the whole field deformation in a head and neck case relies upon 1000–2000 reliable corresponding anchor points. Deformable propagation of the ROIs is then achieved by applying the TPS to the triangular vertices of the ROI mesh.

### Evaluation of ROI propagation

The DIR algorithms were tested on clinical head and neck helical kilovoltage (kV) CT data sets taken of 22 patients for the purposes of ART. Patients were being treated for a range of head and neck neoplasms in the oropharynx, nasopharynx, larynx, nasal cavity and paranasal sinus and oral cavity. Each data set consisted of a contrast-enhanced CT scan taken at the time of planning and a second contrast-enhanced CT scan taken between 11 and 35 days into the treatment. Image slice thickness varied from 2 to 3 mm. Both CT scans had the following ROIs delineated by an experienced physician at each institution: spinal cord, brainstem, parotid glands and gross tumour volumes (GTVs). On two patients the right parotid was not delineated due to the location of the GTV. Rigid translation and rotation registration was performed using a cross-correlation algorithm followed by DIR between the image sets. The resultant deformation maps were then applied to the ROIs corresponding to the first CT image to result in a set of ROIs corresponding to the second CT image. The DIR-created ROIs were then compared with the expert-contoured ROIs using the Dice volume overlap score (DS) [20] and the mean of the slicewise Hausdorff distances (MSHD). The MSHD was obtained by calculating the symmetric Hausdorff distance [21] on each slice, and taking the mean of this over all slices containing expert-contours. The DS for two ROIs A and B is defined as DS = 2|A∩B|/(|A| + |B|), where |*X*| is the number of voxels enclosed by ROI *X*. Additionally, for the GTV, the center-of-mass (COM) displacement vector of each DIR-propagated GTV ROI from the expert-drawn GTV ROI on the target image was measured. The COM displacement for the GTV was investigated as it has implications on the position of the isocenter for replanning. In addition to the above metrics, the time taken to perform DIR between the two images and warp the moving image and all of the ROIs was also recorded. A one-way Analysis of Variance (ANOVA) test was carried out on each set of comparison metric to determine statistical significance, with a threshold of p < 0.05, using Matlab (R2010b, MathWorks, Natick, MA).

Inter and intra observer variations exist in the generation of ROIs [22,23]. The metric comparisons are thus sensitive to these variations in the generation of the ground truth ROIs. Therefore the expert physicians were also asked to score the DIR-propagated ROIs based on the clinical utility of the DIR-propagated ROI. The same physician who drew the original ROIs was used to score the DIR-propagated ROIs, without the assistance of their originally drawn ROI on the per-treatment image. A scoring system of 1, 2 or 3 was used to rate the quality of the propagated ROIs and measure how much editing was required to obtain a clinically acceptable ROI: 1 was given to propagated ROIs that do not require editing; 2 was given to propagated ROIs that require minor edits but are useful; 3 was given to propagated ROIs that require major edits and are not useful.

## Results

### Consistency of ROI propagation

**Figure 1:**
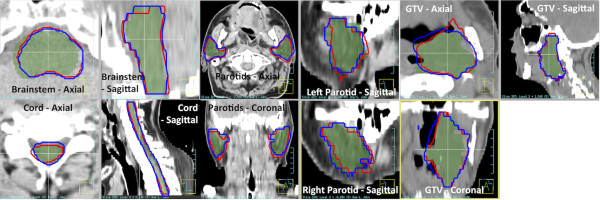
Visual comparison of the expert (green colorwash), Demons-propagated (red contour) and SFBR-propagated (blue contour) ROIs for one patient.

**Figure 2:**
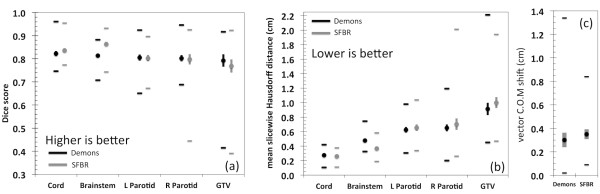
Mean (circles), standard error (vertical lines) and range (horizontal lines) for (a) Dice score, (b) mean of the slicewise Hausdorff distances and (c) COM vector displacement for the GTV.

The DIR-propagated ROIs were compared with the expert-drawn ROIs on each image. Figure 1 shows examples of the DIR-propagated ROIs compared with expert ROIs for each organ. Figure 2 shows the Dice score and MSHD between the DIR-propagated and expert ROIs as well as the COM displacement for the GTVs. The only statistically significant difference between the two algorithms was observed for the brainstem, where the SFBR-propagated ROIs had higher Dice scores and lower MSHDs than the Demons-propagated ROIs (*p* = 0.001 &*p* = 0.002 for Dice scores and MSHDs respectively). For all other organs, no statistically significant differences between the two algorithms were observed. For one patient, a large difference was observed for the right parotid – this is shown by the small minimum Dice and large maximum MSHD for SFBR in Figure 2. This particular patient showed a strong response to the radiotherapy: the external contour, thus the GTV, receded up to 3.5 cm medially on the patient’s right side. The differences in the GTV COM locations are shown in Figure 2c. Although the mean GTV COM shift was lower with Demons, this result was not statistically significant.

The times taken for deformation, image warping and ROI propagation are shown in Table 1. Deformation with SFBR took approximately 55% longer on average (p = 0.009).

**Table 1:** Total deformation time including time to warp the image and ROIs for both algorithms

**Algorithm**	**Average (s) ± SEM (s)**	**Range (s)**
Demons	241 ± 28	110 – 508
SFBR	375 ± 40	109 – 660

**Figure 3:**
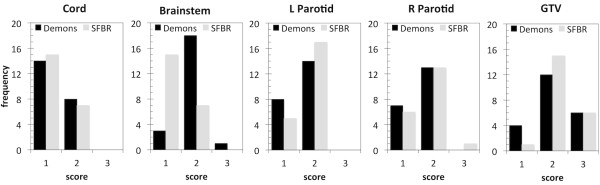
**Histograms of the expert scores of each organ**. 1 = Propagated ROI is fine with no edits; 2 = Propagated ROI requires minor edits, is useful; 3 = Propagated ROI requires major edits, not useful.

Expert physicians scored each of the DIR-propagated ROIs generated for all 22 patients based on the scoring system defined above. Figure 3 shows histograms of the scores for the five organs. The majority of the scores were 1 (n = 78) or 2 (n = 124), with 14/216 ROIs scored 3. Out of the 14 ROIs scored 3, 12 were GTVs, with the other two being a brainstem (Demons) and one right parotid (SFBR), both from the same patient, as mentioned above.

## Discussion

The agreement of DIR-propagated ROIs from the DIR algorithms used in this study with the physician drawn ROIs was shown to be dependent on the organ of interest. The DIR-propagated OARs were generally sufficiently accurate for clinical use with minor or no corrections. DIR propagated target ROIs were found to be less accurate, mainly due to the subjective nature of target definition in adaptive radiotherapy. Propagation of ROIs using DIR took between 2 and 11 minutes, within the realm of clinical utility. Although the two algorithms used in this study are significantly different in their approach to calculating deformation fields, little difference in Dice and MSHD was observed between the two algorithms. Only for the brainstem was there a statistically significant difference in the measures analyzed; SFBR had a higher average Dice score and lower average MSHD meaning better agreement with the physician drawn ROIs.

One observation with the GTV was the difficulty of the SFBR algorithm to accurately determine the air-tissue interface within the pharynx. A large proportion of the GTVs resided on the patient’s airway. A shift in the air-tissue interface in or adjacent to the GTV from the planning to the per-treatmen CT was observed in some patients, due to swallowing, breathing or tumor regression. The use of 4DCT could be employed in these cases to improve target delineation. Figure 1 shows that the Demons algorithm was able to track the air-tissue interface more accurately than the SFBR algorithm, which is expected when one considers that the Demons algorithm is based on iso-intensity contours in the image whereas SFBR is based on point-like feature matching and avoids ambiguities along surfaces. Another observation for many GTVs was that when the repeat scan was obtained several weeks into the treatment, very little of the original tumor remained. As alluded to by other investigators [17], although the GTV (as defined as radiographically apparent tumor) had indeed shrunk, it is likely that there was residual microscopic disease within the original GTV boundary, but outside the GTV as defined on the repeat CT scan. As there is no evidence that these areas do not require the original planned radiation dose, to minimize the risk of local recurrence it is reasonable to use a larger definition more closely related to the original GTV definition. These decisions require clinical judgment, considering anatomic barriers of tumor spread and whether or not an original GTV boundary represents infiltrative tumor (e.g. base of tongue tumor) or rather a “pushing” border with displacement of normal structures (e.g. an encapsulated pathological lymph node). Given the metric and expert physician scores, it is recommended that DIR-propagated GTV ROIs be thoroughly reviewed by the treating physician to ensure adequate target dose coverage for adaptive re-planning [15,17].

**Figure 4:**
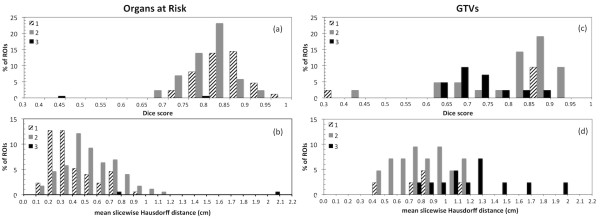
**Histograms of (a) & (c) the Dice scores and (b) & (d) MSHDs for all of the OARs and GTVs grouped into expert scoring category**. The frequencies are normalized to the total number of OARs and GTVs scored in the study (172 and 44 respectively).

Expert physician scores are shown in Figure 3. Although these scores are subjective in that they are based on the opinions of the expert physician, the authors feel that this ultimately represents the clinical utility of the automatically generated ROIs. The scores show that despite some disagreement between DIR-propagated and expert physician-drawn ROIs on the per-treatment CT scans, the majority (202/216 = 94%) of the DIR-propagated ROIs were considered useful and required no or minor changes. The majority of ROIs scored as being not useful or requiring major edits were GTVs. The relationship between the ROI metric scores and the physician scores was investigated. Figure 4 shows histograms of the OARs metric scores grouped into expert physician score category. When comparing only the groups with scores of 1 or 2 with 3 for the OARs, there is a moderate correlation between both Dice score and MSHD and the expert physician score (point biserial correlation r_pb_ = −0.319, p < 0.0001 & r_pb_ = 0.341, p = 0.0001 for Dice scores and MSHDs respectively). For the GTVs, there was no correlation between the Dice and MSHD and expert physician scores (point biserial correlation r_pb_ = −0.002, p = 0.49 & r_pb_ = 0.185, p = 0.17 for Dice scores and MSHDs respectively), however these values are not statistically significant, most likely due to too few GTV samples. Figure 4 suggests that the metrics used in this study have clinical relevance for OARs, but not necessarily for GTVs. This is most likely due to the subjective definition of GTVs by the physician that is based on clinical knowledge and experience rather than pure image intensity values.

The deformation time included the time to pre-process the image (proprietary), perform DIR and warping of the moving image and ROIs. For Demons, this involves dir-ect creation of the DVF, which is then applied to the moving image and ROIs. For SFBR, the process involves the creation of a thin-plate-splines (TPS) map, used to warp the image and the ROIs, followed by the creation of a DVF from the TPS map. Although both algorithms were multithreaded, SFBR had not been optimized for speed. The creation of the DVF and warping of the image volume were not necessary for SFBR contour propagation, but were hardcoded into the algorithm. Removal of image warping and DVF creation decreases the total deformation time. In the case of SFBR, removal of image warping and DVF creation decreases the total deformation time by approximately 70%, showing significant room for efficiency improvement.

## Conclusion

In this study, the clinical acceptability of two DIR algorithms for ROI propagation in head and neck adaptive radiotherapy was shown for OARs. The clinical utility of the DIR-propagated ROIs was assessed by expert physicians, who rated the majority of the propagated OAR ROIs as requiring no or only minor modifications for clinical use. Although there is a role for automatic propagation of target ROIs, it is recommended that DIR-propagated target ROIs be thoroughly reviewed by the treating physician.

## Abbreviations

ROI, Region of Interest; CT, Computed Tomography; OAR, Organ at Risk; GTV, Gross Tumor Volume; ART, Adaptive Radiotherapy; DVF, Deformation Vector Field; COM, Center of Mass; MSHD, Mean Slicewise Hausdorff Distance; DIR, Deformable Image Registration; SFBR, Salient Feature Based Registration; TPS, Thin plate spline.

## Competing interests

Nicholas Hardcastle was funded in part by a grant from Philips Radiation Oncology Systems awarded to Prof. Wolfgang Tomé. Karl Bzdusek and Stéphane Allaire are current employees of Philips Radiation Oncology Systems and Philips Research Medisys, respectively. SA however performed the work for this manuscript whilst employed at Princess Margaret Hospital, ON, Canada.

## Authors’ contributions

NH participated in study design, performed the automatic ROI propagation, collated the results, performed the statistical analysis and drafted the manuscript. WAT participated in study design, analysis of the results and helped draft the manuscript. DMC provided ROI scoring, analysis of the results and helped draft the manuscript. CLB, PWHW, ND, MO, AR assisted with collecting data and helped draft the manuscript. MG, SS, MM, BP provided ROI scoring and analysis of the results. SA, TM, PK and KB wrote the DIR algorithm code, provided assistance with algorithm parameter tuning and participated in study design. All authors read and approved the final manuscript.

## References

[B1] Osorio EMV Hoogeman MS Al-Mamgani A Teguh DN Levendag PC Heijmen BJM Local anatomic changes in parotid and submandibular glands during radiotherapy for oropharynx cancer and correlation with dose, studied in detail with nonrigid registration Int J Radiat Oncol Biol Phys 2008 70 875 882 10.1016/j.ijrobp.2007.10.063 18262099

[B2] Robar JL Day A Clancey J Kelly R Yewondwossen M Hollenhorst H Rajaraman M Wilke D Spatial and dosimetric variability of organs at risk in head-and-neck intensity-modulated radiotherapy Int J Radiat Oncol Biol Phys 2007 68 1121 1130 10.1016/j.ijrobp.2007.01.030 17398025

[B3] Lee C Langen KM Lu W Haimerl J Schnarr E Ruchala KJ Olivera GH Meeks SL Kupelian PA Shellenberger TD MaÒon RR Assessment of parotid gland dose changes during head and neck cancer radiotherapy using daily megavoltage computed tomography and deformable image registration Int J Radiat Oncol Biol Phys 2008 71 1563 1571 10.1016/j.ijrobp.2008.04.013 18538505

[B4] O'Daniel JC Garden AS Schwartz DL Wang H Ang KK Ahamad A Rosenthal DI Morrison WH Asper JA Zhang L Tung S-M Mohan R Dong L Parotid gland dose in intensity-modulated radiotherapy for head and neck cancer: is what you plan what you get? Int J Radiat Oncol Biol Phys 2007 69 1290 1296 10.1016/j.ijrobp.2007.07.2345 17967319 PMC2288571

[B5] Yang S-N Liao C-Y Chen S-W Liang J-A Tsai M-H Hua C-H Lin F-J Clinical implications of the tumor volume reduction rate in head-and-neck cancer during definitive intensity-modulated radiotherapy for organ preservation Int J Radiat Oncol Biol Phys 2011 79 1096 1103 10.1016/j.ijrobp.2009.12.055 20605362

[B6] Ahn PH Chen C-C Ahn AI Hong L Scripes PG Shen J Lee C-C Miller E Kalnicki S Garg M Adaptive planning in intensity-modulated radiation therapy for head and neck cancers: single-institution experience and clinical implications Int J Radiat Oncol Biol Phys 2011 80 3 677 685 10.1016/j.ijrobp.2010.03.014 20619553

[B7] Guckenberger M Wilbert J Richter A Baier K Flentje M Potential of adaptive radiotherapy to escalate the radiation dose in combined radiochemotherapy for locally advanced non-small cell lung cancer Int J Radiat Oncol Biol Phys 2011 79 901 908 10.1016/j.ijrobp.2010.04.050 20708850

[B8] Woodford C Yartsev S Dar AR Bauman G Van Dyk J Adaptive radiotherapy planning on decreasing gross tumor volumes as seen on megavoltage computed tomography images Int J Radiat Oncol Biol Phys 2007 69 1316 1322 10.1016/j.ijrobp.2007.07.2369 17967322

[B9] Wu Q Chi Y Chen PY Krauss DJ Yan D Martinez A Adaptive replanning strategies accounting for shrinkage in head and neck IMRT Int J Radiat Oncol Biol Phys 2009 75 924 932 10.1016/j.ijrobp.2009.04.047 19801104

[B10] Zhang T Chi Y Meldolesi E Yan D Automatic delineation of on-line head-and-neck computed tomography images: toward on-line adaptive radiotherapy Int J Radiat Oncol Biol Phys 2007 68 522 530 10.1016/j.ijrobp.2007.01.038 17418960

[B11] Zhao L Wan Q Zhou Y Deng X Xie C Wu S The role of replanning in fractionated intensity modulated radiotherapy for nasopharyngeal carcinoma Radiother Oncol 2011 98 23 27 10.1016/j.radonc.2010.10.009 21040992

[B12] Al-Mayah A Moseley J Hunter S Velec M Chau L Breen S Brock K Biomechanical-based image registration for head and neck radiation treatment Phys Med Biol 2010 55 6491 6500 10.1088/0031-9155/55/21/010 20959687

[B13] Castadot P Lee JA Parraga A Geets X Macq B GrÈgoire V Comparison of 12 deformable registration strategies in adaptive radiation therapy for the treatment of head and neck tumors Radiother Oncol 2008 89 1 12 10.1016/j.radonc.2008.04.010 18501456

[B14] Sims R Isambert A Grégoire V Bidault F Fresco L Sage J Mills J Bourhis J Lefkopoulos D Commowick O Benkebil M Malandain G A pre-clinical assessment of an atlas-based automatic segmentation tool for the head and neck Radiother Oncol 2009 93 474 478 10.1016/j.radonc.2009.08.013 19758720

[B15] Voet PWJ Dirkx MLP Teguh DN Hoogeman MS Levendag PC Heijmen BJM Does atlas-based autosegmentation of neck levels require subsequent manual contour editing to avoid risk of severe target underdosage? A dosimetric analysis Radiother Oncol 2011 98 373 377 10.1016/j.radonc.2010.11.017 21269714

[B16] Brouwer CL Meertens H Bijl HP Chouvalova O Burlage F Steenbakkers R Langendijk J Veld AVT Computerized re-contouring of H&N organs at risk is a useful alternative to physician re-contouring Radiother Oncol 2010 96 S170 183

[B17] Tsuji SY Hwang A Weinberg V Yom SS Quivey JM Xia P Dosimetric evaluation of automatic segmentation for adaptive IMRT for head-and-neck cancer Int J Radiat Oncol Biol Phys 2010 77 707 714 10.1016/j.ijrobp.2009.06.012 20231063

[B18] Vercauteren T Pennec X Perchant A Ayache N Diffeomorphic demons: Efficient non-parametric image registration Neuroimage 2009 45 Suppl. 1 S61 S72 19041946 10.1016/j.neuroimage.2008.10.040

[B19] Allaire S Pekar V Breen S Hope A Jaffray D Automatic extraction of salient interest points in 3D images for contour propagation in IGRT [abstract] Medical Physics 2008 35 2972

[B20] Dice LR Measures of the amount of ecologic association between species Ecology 1945 26 297 302 10.2307/1932409

[B21] Black PE Hausdorff distance http://www.nist.gov/dads/HTML/hausdorffdst.html%5D

[B22] Brouwer CL Steenbakkers RJHM van den Heuvel E Duppen JC Navran A Bijl HP Chouvalova O Burlage F Meertens H Langendijk JA van't Veld AA 3D Variation in delineation of head and neck organs at risk Radiat Oncol 2012 7 32 10.1186/1748-717X-7-32 22414264 PMC3337234

[B23] Riegel AC Berson AM Destian S Ng T Tena LB Mitnick RJ Wong PS Variability of gross tumor volume delineation in head-and-neck cancer using CT and PET/CT fusion Int J Radiat Oncol Biol Phys 2006 65 726 32 10.1016/j.ijrobp.2006.01.014 16626888

